# Evolution and Degradation Patterns of Electrochemical Cells Based on the Analysis of Interfacial Phenomena at Li Metal Anode/Electrolyte Interfaces

**DOI:** 10.1021/acs.jpcc.5c04292

**Published:** 2025-08-07

**Authors:** Carlos H. Guerrero Navarro, Perla B. Balbuena

**Affiliations:** Department of Chemical Engineering, Department of Chemistry, Department of Materials Science and Engineering, Texas A&M University, College Station, Texas 77843, United States

## Abstract

In this work, we report the results of a theoretical–computational analysis of the solid electrolyte interphase (SEI) growth and degradation dynamics occurring in lithium metal batteries during cycling. We use ab initio-kinetic Monte Carlo simulations to generate a synthetic data set, which is analyzed by machine learning methods. We aim to determine: (i) how modifications in interfacial interaction energies between solid electrolyte interphase (SEI) blocks and between Li ions and SEI facets impact the Coulombic efficiency (CE) of the battery and (ii) what factors, including reactions, microscopic transport, and other interfacial events, may lead to cell performance “failure” during prolonged charge and discharge cycles, signaled as a sharp decay in the CE over cycling. The demonstration of our approach is done on a cell including a Li metal surface interfacing with a previously introduced state-of-the-art electrolyte, and the idea can be applied to any electrochemical system. Outcomes include the identification of the leading chemical, physical, and structural variables causing cell failure and relating them to the electrolyte formulation, thus paving the way to future more refined analysis and electrolyte design.

## Graphical abstract

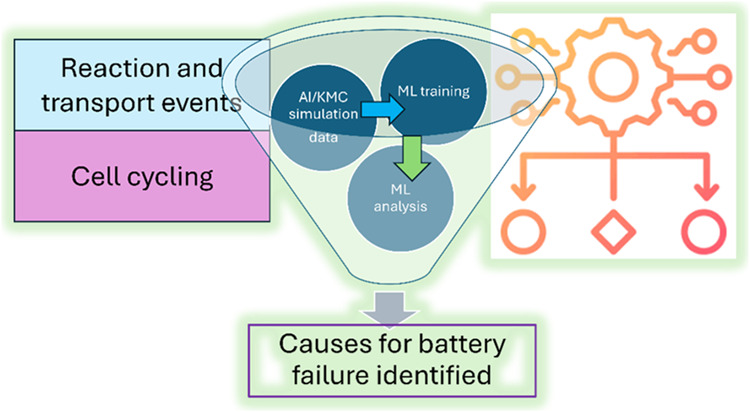

## Introduction

Li metal anodes are among the most promising negative electrodes for rechargeable batteries, which is due to lithium having the lowest electrochemical potential (−3.04 V vs standard hydrogen electrode) and an extraordinarily high specific capacity (3860 mA h/g).^1^ However, mainly because of Li very high reactivity, practical implementations of Li metal batteries encounter numerous challenges,^2^ including dendrite formation, solid electrolyte interphase (SEI) films with undesired chemical and physical properties, and cross-talk events with various cathode materials, all these leading to short battery lifetimes, cumulative degradation events, and catastrophic failure translated in a sudden drop in the specific battery capacity during cycling.^3^ Various strategies to mitigate these issues include electrolyte design, artificial SEIs, anode alloying, or combining with other materials to enhance chemical, electrochemical, and/or mechanical stability.^4^

A crucial topic that may help elucidate battery degradation behavior is related to the interfacial phenomena taking place at the anode/electrolyte interface, including SEI formation, growth, and evolution, along with simultaneous Li plating, stripping, as well as ionic and molecular diffusion.^5^ Advances in current surface and interfacial experimental characterization methods allow elucidating both SEI formation and Li evolution morphologies during cycling.^6,7^ First-principles, atomistic-based modeling at various time/length scales has shown to provide additional insights that enhance fundamental understanding and can offer fresh design principles targeting not only high but also sustained battery capacity.^8^

Our recent study^9^ introduced a novel ab initio-kinetic Monte Carlo (AI-kMC) algorithm to elucidate simultaneous Li electrodeposition phenomena with SEI formation, growth, and evolution, during battery cycling. The model can simulate interfacial phenomena during battery cycling for hundreds of hours. The analyses yielded two important conclusions: (1) The SEI forms in two basic stages: a fast initial growth where SEI blocks (organic and inorganic) are nucleated and a slower aggregation stage where the basic SEI blocks may agglomerate, forming an interphase with chemical and physical features depending on the electrolyte chemistry and environmental variables (e.g., applied voltage, current rate); (2) Li electrodeposition phenomena (plating and stripping) strongly depend on the SEI structure and changes during cycling. Bonding energies of Li ions to SEI facets and interfacial energies between SEI blocks are key elements to a stable plating. During charge, these “channels” would allow (or not) Li ions to get to the surface and become reduced during plating, and during discharge, after oxidation, Li ions would be able to travel back to the electrolyte phase (stripping). The data generated by AI-kMC during a complete charge/discharge cycle are huge and grow with every cycle, and the information contained in these trajectories has great potential for further analysis and conclusions. On the other hand, machine learning (ML) approaches have appeared as significant helpers for data analyses,^10^ including an increasing number of applications to battery research.^11–15^

Thus, we combined AI-kMC and ML methodologies to analyze and extract new information related to battery degradation and the factors that may cause battery failure. We report a detailed analysis of results from a theoretical–computational study of SEI growth and degradation dynamics occurring in lithium metal batteries during cycling. We use AI-kMC simulations^9^ to generate a synthetic data set for a Li metal/electrolyte system that we studied in detail in previous work.^16^ The data set is used in part for training and in part for testing of machine learning (ML) models for predicting factors affecting the Coulombic efficiency (CE), taken as a measurement of the battery decay. We analyze how changes in interfacial interaction energies between Li ions and SEI blocks and between SEI blocks impact the battery CE over cycling and elucidate the main chemical and environmental factors leading to cell performance “failure,” signaled as a sharp decay in the CE over prolonged cycling. By detecting specific interfacial microscopic events (reactions, diffusion) and identity of SEI chemical species and their mobility properties most likely to induce cell failure, we relate this knowledge not only to the electrolyte formulation but also to the cell level, thus facilitating future more refined electrolyte design and battery operation features.

## Computational Methods

The methodology in this research uses data generated by AI-kMC simulations,^9,16^ emulating the electrochemical reactions and diffusion events occurring in a simulated electrochemical cell cycling between charge and discharge. The potential energy surfaces and activation barriers used in the AI-kMC model were obtained from first-principles methods such as density functional theory (DFT) and ab initio molecular dynamics (AIMD). The electrochemical performance in the simulated electrochemical system is defined by the calculated CE at each discharge–charge cycle obtained from AI-kMC simulations. Based on the generated data, ML algorithms are employed to characterize factors that affect the CE, using ML techniques such as SHAP explanatory analysis^17^ and Bayesian optimization.^18^ The electrochemical system investigated^9,16^ consists of a Li metal anode slab in contact with an electrolyte solution of composition 1.2 M lithium bis­(fluorosulfonyl) imide salt (LiFSI) dissolved in a 6.5 M fluorinated ether solvent: 1-(2,2-difluoroethoxy)-2-(2,2,2-trifluoroethoxy) ethane (F5DEE). The DFT calculated electrolyte decomposition mechanisms are depicted in Figure 1.^9,16^ Initially, we used our AI-kMC algorithm, which models a series of chemical and electrochemical microscopic events occurring at the anode side during battery cycling, including electrolyte decomposition reduction reactions, adsorption and diffusion of all involved species governing the initial growth and aggregation of SEI blocks of individual chemical organic and inorganic species, and the plating and stripping reactions of Li ions arriving and leaving the metal anode surface, respectively, at each cycle. The cathode is not explicitly modeled; however, the electrolyte is cycled between the anode and the cathode during each charge–discharge cycle.

**Figure 1:**
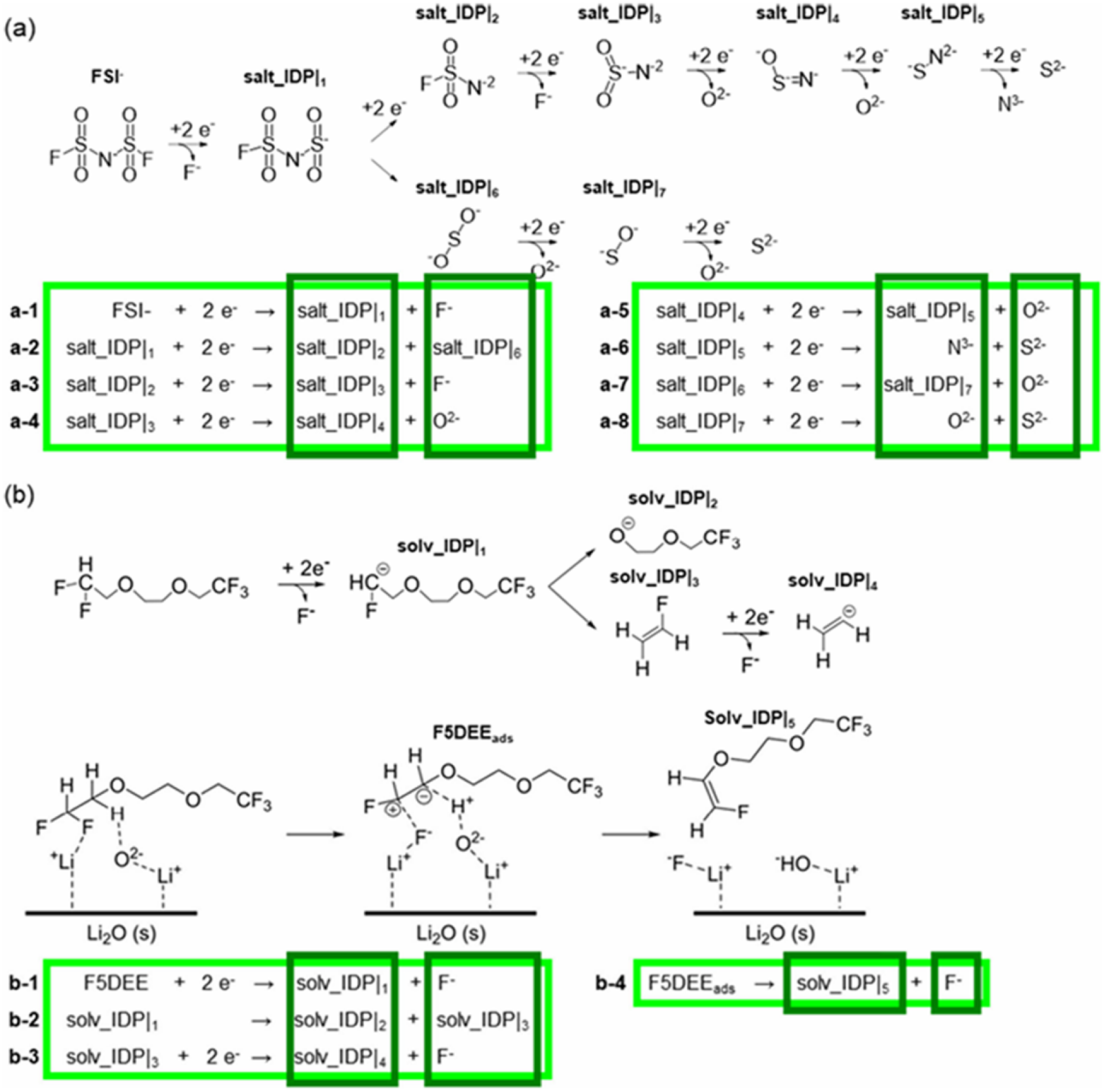
Reaction decomposition pathways for the (a) LiFSI salt and (b) F5DEE solvent on lithium metal. The diagram presents a series of elementary reactions highlighted in light green that drive the complete reduction of each molecule into its constituent species involved in SEI formation and growth. Intermediate products and elemental fragments, shown in dark green, represent the species involved in diffusion processes during the SEI’s aggregation and densification stages. Image reproduced with permission from ref 9 Copyright 2024 American Chemical Society.

All of these microscopic events are represented by simplified kinetic models based on the potential-dependent Arrhenius equation (eq 1). The reaction rate constants are defined by

1 $$k_{i}^{j}=\nu e^{-E_{i}^{j}+\alpha_{i}^{j}\left(E-E_{0,i}^{j}\right)/k_{B}T}$$

where *k*_*i*_^*j*^ is the rate constant for a given *i*th species in reaction *j*th, ν is the frequency parameter, and *E*_*i*_^*j*^ denotes the energy barrier, calculated by DFT and AIMD in previous studies.^9^ α_*i*_^*j*^ is a transfer coefficient, *E* is the applied potential, *E*_0,*i*_^*j*^ represents a reference potential, *T* is the absolute temperature, and *k*_B_ is the Boltzmann constant. The selection of events and their occurrence times were determined by the First Reaction Method,^9,19^ and the random numbers were obtained by the pseudorandom number generator (PRNG),^20^ in order to ensure an accurate representation of microscopic dynamics of the SEI. Table 1 displays the nomenclature used to characterize the main SEI constituents. Thermodynamic and kinetic parameters used in these simulations are provided in the Supporting Information.

**Table 1:** Nomenclature Used to Identify the Main Reaction Products According to Reactions in Figure 1

name	meaning
FSI^–^	bis(fluorosulfonyl) imide anions
Li	lithium atoms in the cell (include SEI and anode surface)
F^–^	fluoride anion produced in salt decomposition reactions a1 and a3, and solvent decomposition reactions b1, b3, and b4
F5DEE	solvent adsorbed over the Li surface or Li_2_O SEI surface.
N^3–^	nitride anion from decomposition of salt_IDP5 (reaction a6)
O^2–^	oxide anion produced by decomposition of intermediate products salt_IDP3, salt_IDP4, salt_IDP6, and salt_IDP7 (reactions a4, a5, a7, and a8)
S^2–^	sulfide ion resulting from decomposition of salt_IDP5 and salt_IDP7 (reactions a6 and a8)
SOL	solvent decomposition products: solv_IDP1, solv_IDP2, solv_IDP3, and solv_IDP4 (reactions b1, b2, b3)
SFO^–^	salt decomposition products: salt_IDP1, salt_IDP2, salt_IDP3, salt_IDP4, salt_IDP5, salt_IDP6, and salt_IDP7.

Table 2 displays the DFT values of a group of the main pair-bonding interactions between atomic pairs, atom–fragment, or fragment–fragment species for the most relevant pairs in the system of study. Note that interfacial ionic mobilities are calculated from the DFT pair-bonding energies calculated at a given interface.^9^ We conducted 380 AI-kMC simulations. In each simulation, we varied 19 pair-bonding interaction energies (listed in Table 2) within the range of 0 to 1.5 eV, using fixed increments of 0.1 eV. In the first set, each interaction was modified individually (one at a time), while in the random simulation set, multiple interaction energies were changed simultaneously in a random manner, still maintaining the 0.1 eV increment step for each. The rationale behind this design is 2-fold: the fixed 0.1 eV increments allow us to isolate and observe the effect of each interaction in a controlled manner, while the randomized multivariable simulations better reflect the complex and correlated variations likely to occur in realistic systems, where several bonding environments evolve simultaneously.

**Table 2:** Pair-Bonding Interaction Parameters from DFT Calculations^9^

atomic pair	pair interaction energy (eV)
Li–Li	0.1037
Li–F	0.7146
F–F	0.0337
F–SOL	0.5000
SFO–Li	0.7146
SFO–SFO	0.0337
SFO–SOL	0.7500
O–Li	0.8027
O–O	0.0615
O–SOL	0.5000
F5DEE–Li	0.7146
F5DEE–F5DEE	0.0337
F5DEE–SOL	0.7500
S–Li	0.7439
S–S	0.0735
S-SOL	0.5000
N–Li	0.9000
N–N	0.1166
N–SOL	0.5000

Each AI-kMC simulation was run for 100 cycles of charge and discharge (switching the voltage between 4.4 and 2.8 V, respectively) with the given electrolyte formulation, at a constant temperature of 298 K.^16,21^ The structural framework is formed by a tetragonal simulation cell, whose dimensions are (40*a*_0_) × (40*a*_0_) in the *x* and *y* horizontal axes and (160*a*_0_) in the vertical *z* direction, where *a*_0_ is the lattice parameter of 4.47 Å. A five-layer lithium slab occupies the center of the cell and is repeated periodically in the *x* and *y* directions. Electrolyte molecules are placed at random on both exposed (001) surfaces of the slab. Periodic boundary conditions are applied in all three directions. Using *a*_0_ = 4.47 Å allows the crystal to accommodate either FCC or BCC like motifs, reproducing the range of Li^+^ coordination environments that occur at the Li/SEI interfaces.

From every simulation, we extracted the Coulombic efficiency (CE) using the following definition^22^

2 $$\mathrm{CE}=\left(\frac{Q_{\mathrm{dis}}}{Q_{\mathrm{ch}}}\right)100$$

where *Q*_dis_ is the number of electrons exchanged in the discharge of the battery and *Q*_ch_ is the number of electrons in the charge step.

According to the reaction mechanisms displayed in Figure 1, specific chemistry features include the adsorption and decomposition of the solvent and salt anion, plating and stripping of lithium over the surfaces (anode and SEI), and SEI formation of various ions (N^3–^, S^2–^, O^2–^, and F^–^). Other events involve the evolution of species such as Li buried (inactive Li trapped among SEI blocks) and Li ions present on the surface (diffusing or forming SEI), and mobility of individual species *M*_*t*_^*j*^ that result from counting each chemical species per layer and cycle. The mobility of a given species *j* is calculated by

3 $$M_{t}^{j}=\sum_{i=1}^{L}\left|m_{i}^{j}\left(t+1\right)-m_{i}^{j}\left(t\right)\right|-G_{t}^{j}\left(t\right)$$

where *M*_*t*_^*j*^ corresponds to a mobility rate for species *j*th, in the cycle *t*th, *m*_*i*_^*j*^(*t*) and *m*_*i*_^*j*^(*t* + 1) are the net amounts of the species *j*th in the layer *i* and cycle *t*th, for the charge and discharge steps, *G*_*t*_^*j*^(*t*) is the total amount of species *j*th produced by reaction in the cycle *t*th, and *L* is the number of cell layers.

The synthetic data set based on the 380 kMC simulations (with more than 35,000 events) was cleaned, eliminating noise and outliers (see the Supporting Information for details of the filtering rules), normalized, and labeled. The data set was divided into 70% for the training process and 30% for the test, following a typical distribution ratio used in materials informatics work.^23^ The training set was used to fit and tune a wide range of ML methods, such as classical regression methods: Linear, Ridge and Lasso Regression, SVR, Random Forest, Extra Trees, Gradient Boosting, XGBoost, and CatBoost.^24–31^ Furthermore, we tested neural networks with complex architectures, such as Dense Neural Networks,^32^ 1D Convolutional Neural Networks (CNN),^33^ and Long Short-Term Memory (LSTM);^34^ each model was optimized using the Adam algorithm,^35^ with the activation function ReLU,^36^ and trained by loss function root mean squared error (RMSE).^37^ From the various ML methods employed, we selected the best model using statistical metrics, such as RMSE, coefficient of determination (R^2^), and mean absolute error (MAE). Performances of the models were evaluated using k-fold cross-validation to ensure robustness and minimize overfitting.^38^ This methodological approach aligns with recent advancements in the field, where ML models have been applied to predict critical properties of electrolytes and materials, like ionic conductivity, Coulombic efficiency, and specific capacity.^39–42^

The SHapley Additive exPlanations (SHAP) method was utilized to interpret the results of the best models selected in the previous step. SHAP assigns each feature an importance value for a particular prediction ϕ_*i*_, known as the Shapley value, expressed as^43^

4 $$\phi_{i}=\sum_{S\subseteq N\backslash }\frac{\left|S\right|!\left(\left|N\right|-\left|S\right|-1\right)!}{\left|N\right|!}\left[f_{S\cup \left\{i\right\}}\left(x_{S\cup \left\{i\right\}}\right)-f_{S\left(\mathrm{xs}\right)})\right]$$

where *N* is the set of all features, *S* is a subset of features not containing *i*, and *f*_*S*(*xs*)_ denotes the model trained with features in subset *S*, evaluated at the corresponding values *x*_*s*_. In this study, the SHAP method allows us to evaluate, for every simulation, how much each input variable, such as those in Tables 2 and 3, may change the predicted Coulombic efficiency.

**Table 3:** MSE, RMSE, and *R* ^2^ of the Best Models Tested

model	RMSE	MSE	*R* ^2^
DenseNN	1.9284	3.7188	0.9881
MLPRgressor	2.1401	4.5800	0.9861
XGBoost	3.2812	10.7661	0.9673
Ridge	3.6520	13.2143	0.9599
Lasso	3.6497	13.3204	0.9595
Extra Trees	3.6960	13.6607	0.9585
Random Forest	4.0675	16.5447	0.9497
Gradient Boosting	4.1294	17.0523	0.9482
LSTM	4.6208	21.3516	0.9391
CatBoost	4.7158	22.2391	0.9325
DecisionTree	6.2694	39.3049	0.8806
SVR	7.7047	59.3624	0.8197
CNN1D	8.8218	77.8242	0.7412

Bayesian optimization (BO)^44^ was employed to identify optimal configurations of the most important pair-bonding interactions identified previously by SHAP. BO was implemented in Optuna^45^ using its Tree-Structured Parzen Estimator (TPE) sampler, which builds a nonparametric surrogate model of the objective function at each iteration. Gaussian Process (GP) has been successfully tested for nonparametric and flexible models in lithium battery systems.^11^ The study was scheduled for 150 trials but converged in approximately 40 evaluations, making it computationally efficient. These optimal values were compared with the theoretical ones from the DFT predictions^9^ shown in Table 2.

Furthermore, a deep analysis regarding the battery “failure” was made using the same synthetic data set. This part of the analysis was divided into 2 parts. The first one consisted of the study of the same reaction, mobility, and morphology evolution features at intervals of specific CE. At each interval, every parameter was compared using classical statistical measures (standard deviation, average, variance, etc.) to observe possible changes that may cause a decrease in the CE. The results were embodied in heatmaps to facilitate the interpretation. In the second part of the analysis, we divided the simulation results into failure and nonfailure regions, and, similar to the first part, we compared both regions to detect possible changes that may cause drops in the CE. Moreover, since not all combinations of every pair-bonding interaction lead to battery failure before 100 cycles, we trained several binary classifiers, including Logistic Regression,^46^ Random Forest,^47^ Gradient Boosting,^29^ CatBoost,^48^ and XGBoost.^30^ We evaluated the model’s performance using metrics such as accuracy, precision, recall,^49^ F1-score,^50^ ROC curves^51^ and AUC values,^52^ in order to select the best model. These methods have been successfully applied in similar battery degradation studies, for instance, Wang et al.^53^ used multiple classifier methods to accurately predict the battery end-of-life for commercial lithium iron phosphate/graphite cells. Once the optimal classifier was identified, we employed the SHAP method to find which pair-bonding interaction contributes to premature failure.

Both analyses provide new insights regarding the evolution and degradation of the SEI in lithium metal batteries, offering an example of the development of robust predictive tools to guide the design and optimization of future advanced battery-based energy storage technologies.

## Results and Discussion

### ML Model Selection

Figure 2 and Table 3 show that the best-performing models according to the metrics RMSE, MSE, and *R* ^2^ are, in descending order, 4 layers DenseNN (512, 256, 128, and 1 neurons), MLPRregressor, XGBoost, and Extra Trees. The correlation coefficient of DenseNN is close to 1, which means an excellent fit with the data set. Furthermore, the parity plots in Figure 2 confirm a good fit and nonoverfitting, since the points are homogeneously distributed along the 1:1 line, with accumulation near 100% CE.

**Figure 2:**
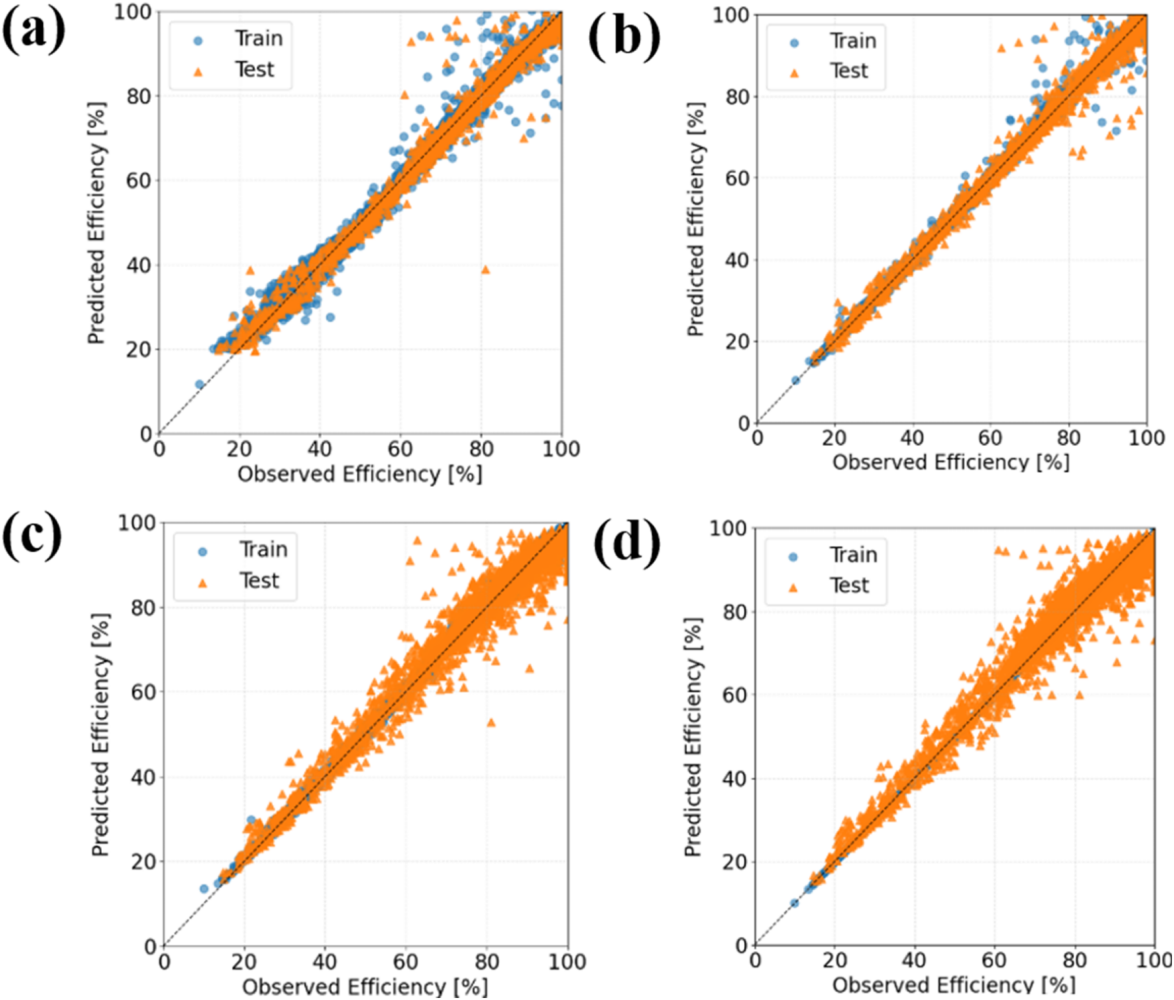
Predicted vs observed CE according to four models: (a) Dense Neural Network, (b) MLPRegressor, (c) XGBoost, and (d) Extra Trees achieve nearly perfect training fits; the Dense Neural Network exhibits the smallest prediction errors and tightest test-set clustering, followed closely by the MLPRegressor; XGBoost performs well with a slightly higher variance at high CE.

Figure 2 shows that while all four models—Dense Neural Network, MLPRegressor, XGBoost, and Extra Trees—achieve nearly perfect training fits, the Dense Neural Network exhibits the smallest prediction errors and tightest test-set clustering, followed closely by the MLPRegressor; XGBoost performs well with a slightly higher variance at high CE.

### Effects of Relevant CE Features from Predictions of the Selected ML Model

Figure 3 shows a beeswarm plot of the SHAP values for the most important interfacial features in the system of study obtained from the DenseNN model. The horizontal SHAP value is expressed in percentage points of CE, where 0 means the average value of CE. Each dot is one simulation, colored red when the feature value is high and blue when it is low. Thus, a red (blue) cluster far to the right signals that larger (lower) values of that variable consistently improve CE, whereas a red (blue) cluster to the left shows that higher (lower) levels harm it. A dense cluster occurs when many simulations share the same feature value, so their dots overlap; a feature can therefore look as if it has only a few points even though it is present in every record. If a feature’s dots are close to 0 value, it means that the feature has a negligible effect on the objective variable prediction (CE). The horizontal SHAP axis uses the same units as the objective variable (percentage points of CE), so each SHAP value represents how much that feature shifts the predicted CE for a given simulation. Dense clusters of points arise when a feature takes nearly identical values across many simulations, which means that their SHAP contributions are almost the same and thus overlap visually, whereas a spread of points indicates that the feature varies widely and produces a broader range of impacts. Sometimes, a feature appears to have very few points, not because it is missing data, but because it only yields a small set of unique values, causing many overlapping points to merge into what looks like a single marker. Features are ranked in descending importance order from top to bottom.

**Figure 3:**
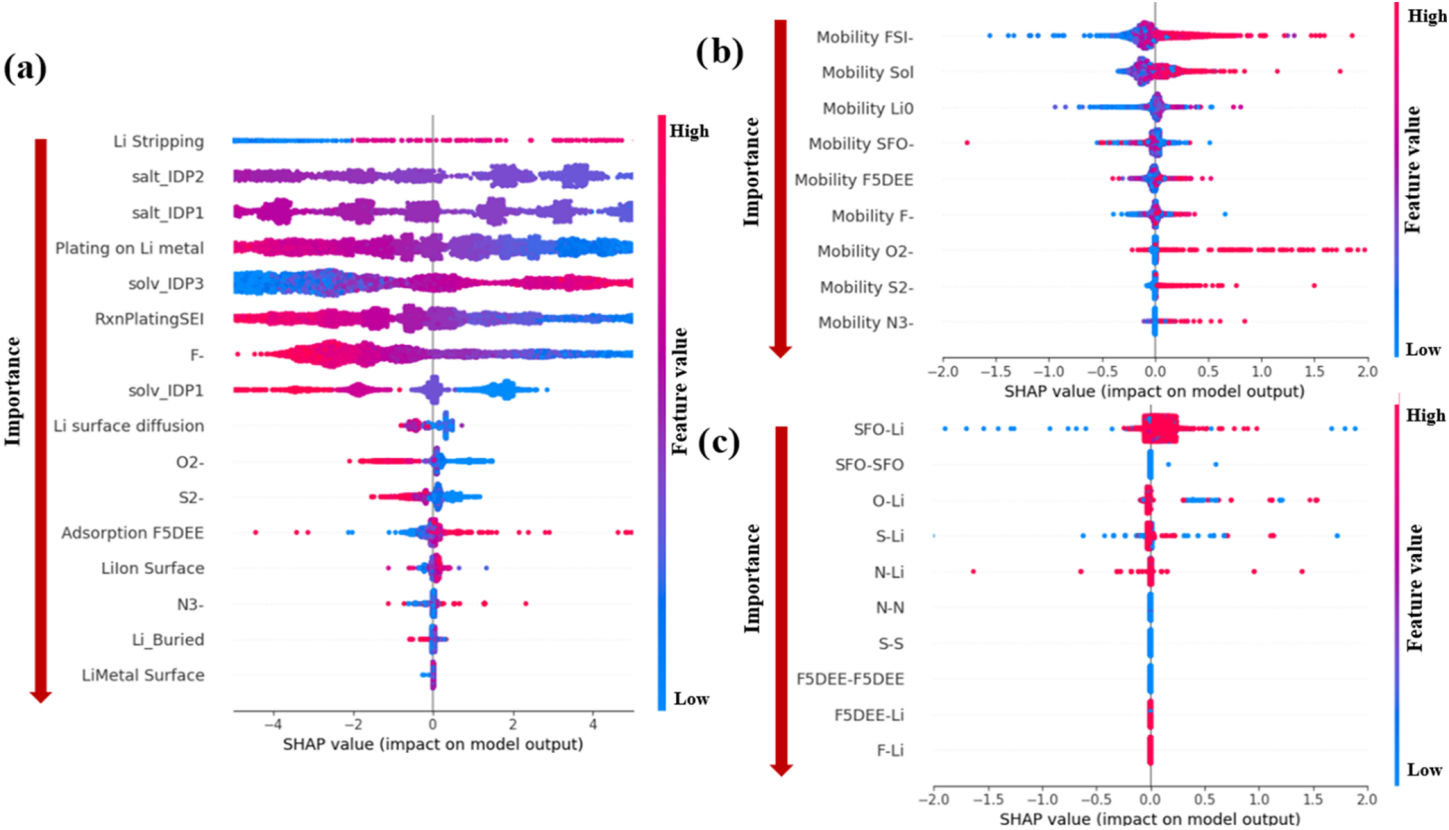
SHAP analysis from the Dense Neural Network model. (a) Chemical process features where Li stripping, solv_IDP3, and salt_IDP1 are the strongest positive drivers of CE, while salt_IDP2 and N^3–^/S^2–^ formation negatively impact it. (b) Mobility parameters for FSI^–^, F5DEE, and SFO correlate positively with CE, whereas excessive Li^0^ or S^2–^ mobilities are detrimental. (c) Most critical pair-bonding interactions, indicating that optimum Li-X interaction values are small.

Figure 3a shows that Li stripping is the most important parameter, which is chemically consistent, since stripping is the main contribution to the discharge reaction, the term *Q*_dis_ (eq 3). According to our simulations, the amount of the F5DEE solvent decomposition product (solv_IDP2) is too small; thus, it can be disregarded (as shown in Table 4), consistent with the NMR operando results of May et al.,^54^ which reveal that late-stage fragmentation of fluorinated ether is kinetically limited under practical current densities. Figure 3a also reveals a clear positive impact to the CE from the solvent decomposition intermediate product vinyl fluoride (solv_IDP3), but a clear negative impact (but less important due to its hierarchy in the vertical order of SHAP values) for the early solvent decomposition product 1,1,1-Trifluoro-2-[2-(2-fluoroethoxy) ethoxy] ethane (solv_IDP1), suggesting that the presence of high concentrations of solv_IDP3 tends to improve the battery performance. This means that the CE would be favored if the solvent is further decomposed beyond solv_IDP1 and remains as solv_IDP3 without further defluorination.

**Table 4:** Parameters Used to Train the Model are Summarized through Key Descriptive Statistics^a^ [Table tbl1]

parameter	mean	Std	min	25%	50%	75%	max
CE	81.077	17.680	10	75.385	85.714	94	100
Li stripping	42.509	10.924	6	43	48	49	50
salt_IDP2	8.329	6.772	0	3	7	12	36
salt_IDP1	8.728	4.891	0	5	9	12	28
solv_IDP3	10.546	7.325	0	4	11	16	42
solv_IDP2	0.012	0.00042	0	0	0	0	1
Plating on Li metal	8.233	4.911	0	4	8	12	29
RxnPlatingSEI	7.818	3.777	0	5	8	10	22
F^–^	21.383	6.865	0	17	22	26	45
solv_IDP1	1.052	1.111	0	0	1	2	7
Adsorption F5DEE	12.638	7.139	–1	7	13	18	43
S^2–^	2.079	2.114	–8	0	2	3	13
O^2–^	4.104	3.669	0	1	3	6	22
N^3–^	1.053	1.290	–3	0	1	2	10
Li Ion Surface	2.156	33.035	–133	–16	6	23	144
Li Buried	63.920	37.692	–518	44	65	86	303
Li metal Surface	201.018	55.094	–89	168	208	239	548
Mobility F^–^	17.336	19.613	0	3	12	27	213
Mobility F5DEE	14.267	18.817	0	1	7	23	191
Mobility FSI^–^	72.852	43.332	0	34	83	109	204
Mobility Li0	61.236	36.507	0	33	54	83	286
Mobility N^3–^	0.161	1.120	0	0	0	0	24
Mobility O^2–^	0.492	2.425	0	0	0	0	31
Mobility S^2–^	0.329	1.964	0	0	0	0	60
Mobility SFO^–^	13.236	16.555	0	2	9	20	185
Mobility SOL	168.979	99.383	0	80	191	252	522

a Mean, standard deviation, minimum, maximum, and 25th, 50th, and 75th percentiles. Mobility is quantified as the number of molecules undergoing positional changes per charge/discharge cycle, whereas the other parameters denote the number of reactions or events occurring within each cycle. Variables are defined in Figure 1 and Table 1.

This takes us to the next point, which is the presence of LiF, a well-known and, in many cases, desired SEI component. Interestingly, the results in Figure 3a indicate that the F^–^ generation, which would result in fluorinated compounds starting with LiF, yields an impact on the CE that changes from positive to negative as the concentration of LiF increases. At short times, LiF comes from salt defluorination; however, at longer times, the amount of LiF may increase considerably for this electrolyte due to F5DEE decomposition (reactions b1, b3, b4, Figure 1). Therefore, the SHAP analysis implies that deep F5DEE fragmentation decreases CE. This could be explained because excessive LiF concentrations may increase the ionic resistance, limiting the Li^+^ transport, augmenting the interface polarization, and promoting lithium dendrite growth.^55,56^ On the other hand, Jia et al.^57^ suggested that moderate levels of LiF in SEI improve ionic conductivity and electronic insulation. Furthermore, our results also agree with those of reports of other fluorinated ether solvents. For instance, Zhang et al.^58^ indicated that partial cleavage to fluoro-alkoxy species passivates Li, improving cycling stability and boosting CE and oxidation stability. Lee et al.^59^ indicated that LiF-rich SEI contributes to improved electrochemical performance and reversibility of lithium metal anodes. Additionally, vinyl fluoride (solv_IDP3) could in situ polymerize into poly­(vinylidene difluoride) (PVDF), forming a matrix that ensures an even distribution of lithium, which leads to stable Li^+^ flux.^60^

Exploring the LiFSI decomposition products via SHAP analysis (Figure 3a), we find that the first salt defluorination yielding bis­(fluorosulfonyl)­imide anion (salt_IDP1) shows a positive impact in the low-to-moderate concentration range, which implies that moderate FSI^–^ and LiF coverage fosters a more stable SEI and suppresses the formation of lithium dendrites.^61,62^ By contrast, the presence of the decomposition product fluorosulfonylimide dianion (salt_IDP2) exerts a negative impact on CE. In fact, the negative impact on CE due to the presence of large quantities of salt_IDP2 corresponds to the second most important feature in the model. Thus, according to the mechanism in Figure 1, the product salt_IDP2 should continue reduction, forming further LiF from the second salt defluorination.

All of the previous electrochemical reactions required the presence of e^–^ from the anode to be formed, which by *Q*_ch_ definition reduces CE. Besides F^–^, the further reduction of salt_IDP2 gives products such as S^2–^, O^2–^, N^3–^, which could easily trap incoming Li ions. Note that the SHAP analysis points to S^2–^, O^2–^ negatively influencing the CE. This can be understood based on previous work^9^ showing that Li_2_O reduces the Li^+^ transport due to high interfacial interaction energy (>2.5 eV), which can negatively affect the cell performance. Moreover, according to Jaberi et al.,^63^ the relatively low Li^+^ conductivity in Li_2_O can potentially hinder Li transport during the SEI formation. Also, despite Li_2_S is known for enabling fast Li^+^ transport because of its high conductivity,^64^ some studies^65^ suggest that crystal orientation influences the Li deposition behaviors. Thus, unfavorable Li_2_S crystal facets during SEI aggregation may negatively impact the CE. Li et al.^66^ showed that Li_3_N exhibits high ionic conductivity and thermodynamic stability against lithium metal; its relatively high electronic conductivity (2.6 × 10^–7^ S·cm^–1^ for α-Li_3_N^67^) exceeds the desirable threshold for dendrite suppression. In addition, the low concentration of Li and N vacancies results in limited ionic conductivity and poor resistance to dendrite formation under elevated current densities, ultimately leading to restricted cycling life and diminished capacities.^67^

The results also show that the adsorption of F5DEE over Li_2_O and further decomposition positively impact the CE. This behavior could be explained due to the formation of LiOH adsorbed over Li_2_O (Figure 1), which, according to previous reports, could further react with Li^0^ to form LiH, increasing the SEI stability.^68^ In addition, F5DEE decomposition yields LiF over the Li_2_O surface. The beneficial effect of LiF therefore appears to depend on its provenance, which could explain the positive impact of F^–^ observed in SHAP graphs, as discussed above. Tan et al.,^16^ employing Bader charge analysis, found that the interfacial F5DEE-Li_2_O reaction does not require external flow charge. Furthermore, when analyzing the full range of cycles, F5DEE appears more likely to decompose via the surface adsorption pathway. This is supported by the data in Table 4, where the number of F5DEE decomposition events closely matches the number of LiF formation events, and by Figures S1 and S2, which show that LiF production peaks during the final cycles coincide with a higher frequency of F5DEE adsorption events.

Based on the importance rating, Figure 3a illustrates that lithium plating directly onto the lithium metal surface has a higher impact on Coulombic efficiency than plating onto the SEI. Lower amounts of lithium plating correlate with improved CE, suggesting that maintaining more uniform lithium deposition, preferably on the lithium metal surface, is advantageous. Plated Li can migrate, becoming “buried” or inactive Li that should be minimized according to SHAP results. Other reports suggest that buried or inactive Li could lead to SEI dissolution, dead Li migration into SEI, and Li corrosion, therefore decreasing CE.^69^ According to our results, the presence of Li^+^ ions at the electrode surface is beneficial, as it facilitates uniform lithium deposition and suppresses dendrite formation.^70,71^

Regarding ion mobility, it is widely known that fast Li^+^ transport is necessary for optimal battery performance.^72^ However, the mobility mechanisms of other species within the SEI have not been thoroughly investigated. Our findings, shown in Figure 3b, reveal that once compounds like Li_2_S, Li_2_O, and Li_3_N form and integrate into the SEI, their mobility is relatively low (see Table 4); however, their enhanced mobility could increase the CE, possibly because their redistribution helps form a more uniform SEI. In contrast, LiF exhibits larger mobility during SEI formation, which may allow it to reorganize dynamically within the interphase. This behavior could influence both the mechanical properties and ion conduction pathways of the SEI, indicating a need to increase LiF, Li_2_S, Li_2_O, and Li_3_N mobilities to maintain a more homogeneous interphase. This could be attributed to the fact that composition, thickness, and structure of the SEI are not static after the initial formation,^73^ i.e., during the SEI aggregation stage. Furthermore, Figure 3b shows that the mobility of FSI^–^ anions and solvent molecules (such as F5DEE and its decomposition products) is relevant. The high mobility of these species ensures efficient ion transport and uniform SEI formation and favors the increase of both plating and stripping, which are vital for achieving high CE.^14,15^ However, our SHAP and Bayesian optimization (BO) results also indicate that low mobility of the salt decomposition products (e.g., salt_IDP1 to salt_IDP7) is desirable. When these products migrate excessively, they may disrupt ion pathways or destabilize the SEI. Instead, they should stay in place and react to form the inorganic salt products. Therefore, our findings suggest the following strategy: promote the localized reactivity of salt-derived intermediate species and allow sufficient mobility among key inorganic SEI components (such as LiF, Li_2_O, and Li_2_S) so they can reorganize within the interphase and evolve toward more stable and cohesive structures.

These mobility characteristics are intrinsically linked to the interactions between Li ions and other chemical species. Our analysis indicates that interactions involving lithium have the most significant impact on CE (Figure 3c), whereas interactions among other species have a minimal effect. Specifically, weaker interactions between Li and other components facilitate higher Li^+^ mobility at the respective interfaces, enhancing Li ion transport and overall battery performance, as validated by first-principles computational studies^9^ and results of high ionic conductivity with weak–weak dipole interaction in polymer ionic electrolyte reported by Chen et al.^74^

### Effects of Pair-Bonding Interactions on CE from Bayesian Optimization

To further characterize these pair-bonding interactions and their effect on the CE, we used Bayesian optimization methods shown in Figure 4. The use of Bayesian optimization aligns with the methodology used in battery research. For instance, Noh et al.^12^ and Suzumura et al.^13^ employed BO combined with data-driven methodologies to obtain multiple solvents for a redox-active molecule, 2,1,3-benzothiadiazole, and to achieve the best composition with maximum lithium ion conductivity in a mixture of four LiTFSI solutions, respectively. This validates our multidimensional, data-driven approach as an efficient method to translate complex models into useful experimental guidance.

**Figure 4:**
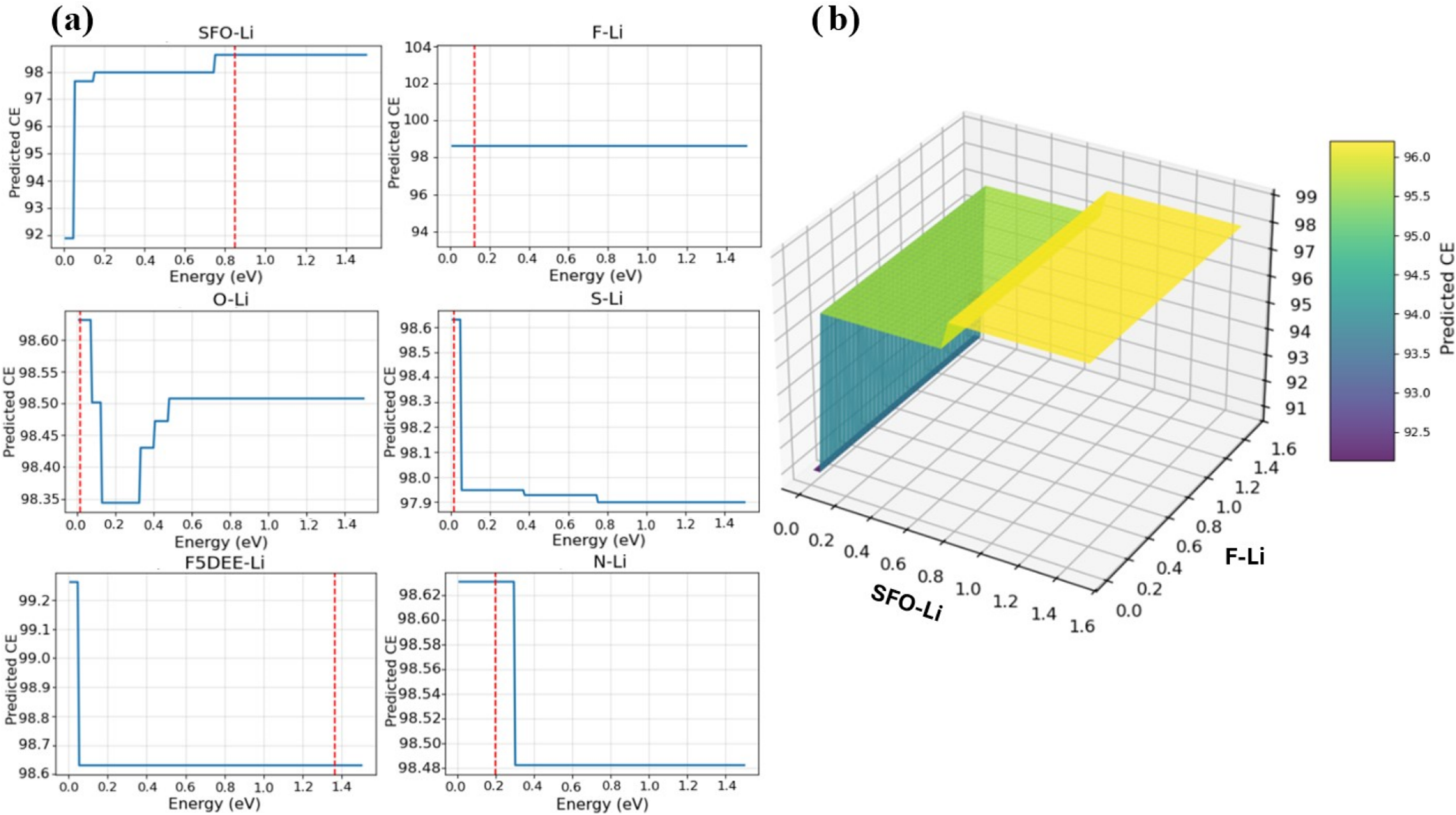
Bayesian optimization analysis for the Dense Neural Network model shows: (a) 2D dimensional plots showing the predicted Coulombic efficiency (CE) as a function of pair interaction energy (in eV) for a few relevant interactions of Li ions with SEI components; (b) 3D plot illustrating the combined effect of SFO–Li and F–Li interactions on the predicted CE.

Bayesian optimization was used to identify the pair-bonding interaction energy strength (measured in eV) that maximizes CE for Li-SEI component interactions, which were detected as the most relevant. The optimization suggests that nearly all bond energies, except those of Li ions linked to salt decomposition products (SFO), should be as low as possible to increase the CE. This aligns with our SHAP analyses, where strong lithium-SEI component interactions reduce battery performance. These optimal pair interaction values can offer useful insights into the design of electrolytes that improve interfacial chemistry. The insights can be summarized into aiming for minimal bonding energies of solvent-derived species and their decomposition products with lithium, while tolerating higher affinities toward salt-derived fragments, to improve battery performance and enhance SEI uniformity. Note that the red vertical line in Figure 4a corresponds to the DFT calculated pair-bonding energy for the respective species. For most inorganic SEI species, the maximum CE agrees with the DFT value. However, during the SEI aggregation process, such a value may vary because the interacting crystallographic facet might not offer the optimum value, and/or because the inorganic species is aggregated with others having different crystallographic characteristics that may change the bonding type.

In Figure 4b, the SFO–Li (anion decomposition products—Li) interaction stood out as the most impactful among all pair energies. Since its optimal value is not near zero, this interaction may be strategically increased, suggesting a targeted avenue for further study. Furthermore, the results indicate that the CE is much less sensitive to specific Li–F interaction values, which makes the LiF features (crystallographic state and type of exposed facet) much more flexible regarding their impact on battery performance. This flexibility may be one of the good properties of LiF as a key SEI component.

### Factors Causing a Sudden CE Drop during Cycling

In the previous sections, we investigated the effects of the various SEI components and their interfacial interactions on the CE, targeting a maximized CE. Another important feature that we aim to obtain from this ML approach is the identification of the factors that eventually cause battery failure, which is characterized by a sudden drop in the CE versus cycle number. Thus, to predict battery failure, we employed two complementary methods. First, we grouped the synthetic data set by intervals of 10% of CE (between 10 and 20%, etc., up to 100% CE) and visualized the normalized feature trends using a heatmap shown in Figure 5 (absolute values are provided in Supporting Information, Figure S3).

**Figure 5:**
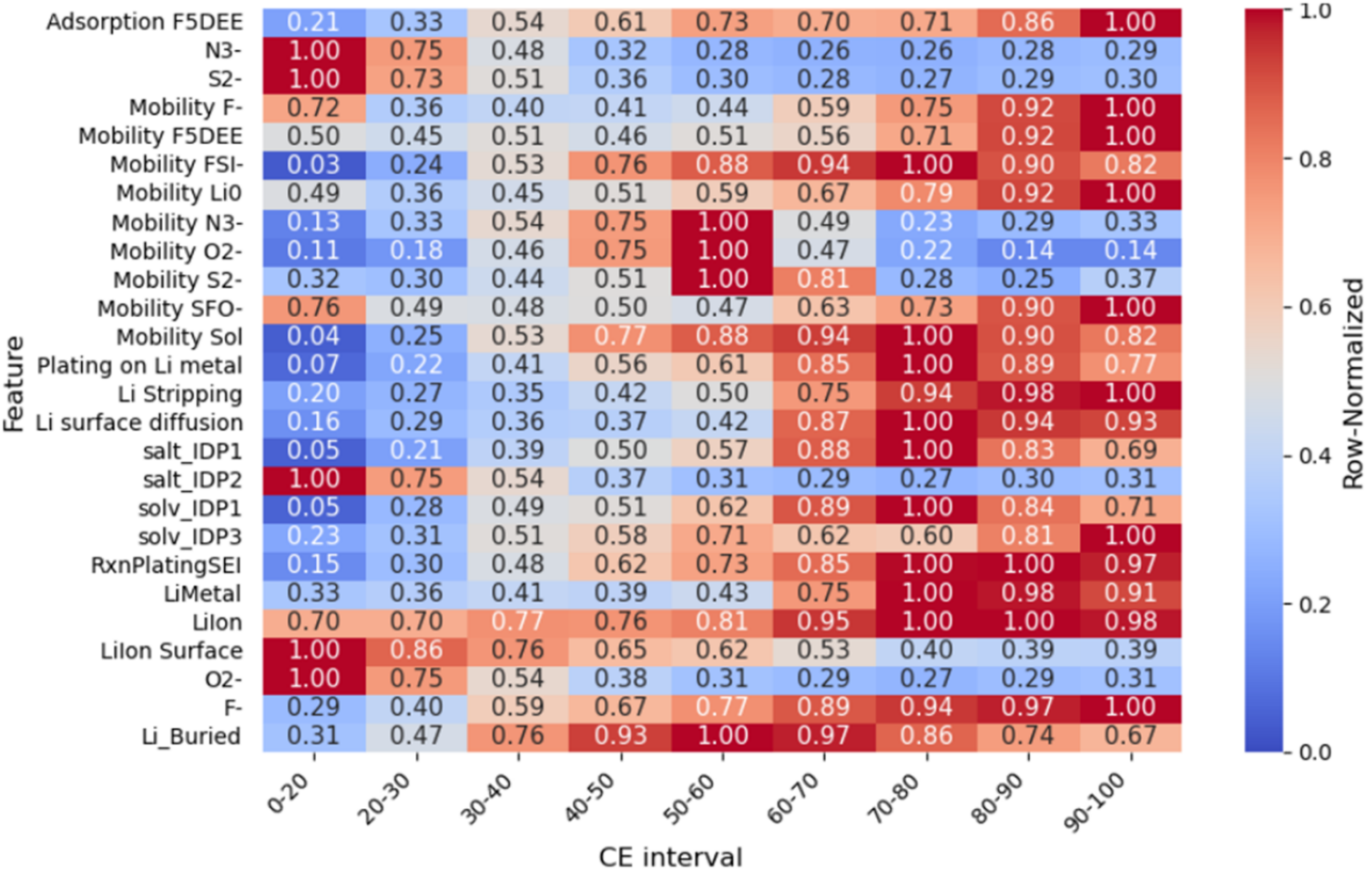
Heatmap of the mean normalized values of each feature, at each of the equal-size CE intervals. Dark red means high normalized values of the given feature, and dark blue means low values. Thus, the heatmap tracks how each feature increases or decreases as CE drops.

This evaluation revealed clear patterns. From the intervals with low CE values (∼<30–40%), it is found that low values (blue boxes) of reactions lead to formation of F^–^ as given by salt and solvent defluorination: salt_IDP1, solv_IDP1, and formation of vinyl species solv_IDP3, F5DEE adsorption, and low mobilities of FSI^–^ and SOL fragments, as well as low plating reactions over SEI and surface, and low Li stripping reactions correlate with decrease of CE. On the other hand, red boxes in the same CE intervals show that the CE fall is strongly correlated with the rise of N^3–^, O^2–^, and S^2–^ species and accumulation of salt_IDP2 (thus impeding second salt defluorination, Figure 1). Other parameters of the same Low CE intervals showed no consistent trend. Likewise, the values for most of the parameters in the high-efficiency band between 70 to 100% present a non-clear pattern, which could indicate that in this region the battery is stable, so below 70% suggests the onset of battery degradation, which aligns with literature criteria.^75,76^ We employed this condition for identifying the main features according to the second approach. The findings are consistent with our earlier SHAP analysis and, combined with the absolute values of the event quantities, could provide approximate magnitudes and specific chemical features signaling the beginning of the battery failure.

In the second method, we categorized all simulations in the data set into two groups: failure and nonfailure based on whether Coulombic efficiency dropped below the threshold defined earlier (70%). We then compared the mean normalized values of all features across both groups using a heatmap, as shown in Figure 6 (the absolute values are presented in Figure S4). This analysis helps us to summarize the results shown in Figure 5. The most important change associated with failure is the sharp decrease (almost 90%) in the mobility of species FSI^–^ and SOL, which reflects the importance of the SEI dynamics in the lithium battery performance. This suggests that loss of mobility of key SEI species may disrupt SEI reassignment during cycling, thus impeding Li ion accessibility at the interface for plating, contributing directly to CE degradation. Therefore, a significant drop is observed in the formation of solv_IDP1 and salt_IDP1, as well as in lithium plating on the metallic surface, which are features previously linked to efficient and reversible lithium cycling. Their reduction may indicate the beginning of interfacial instability or depletion of reactive species that could contribute to a stable and dynamic SEI.

**Figure 6:**
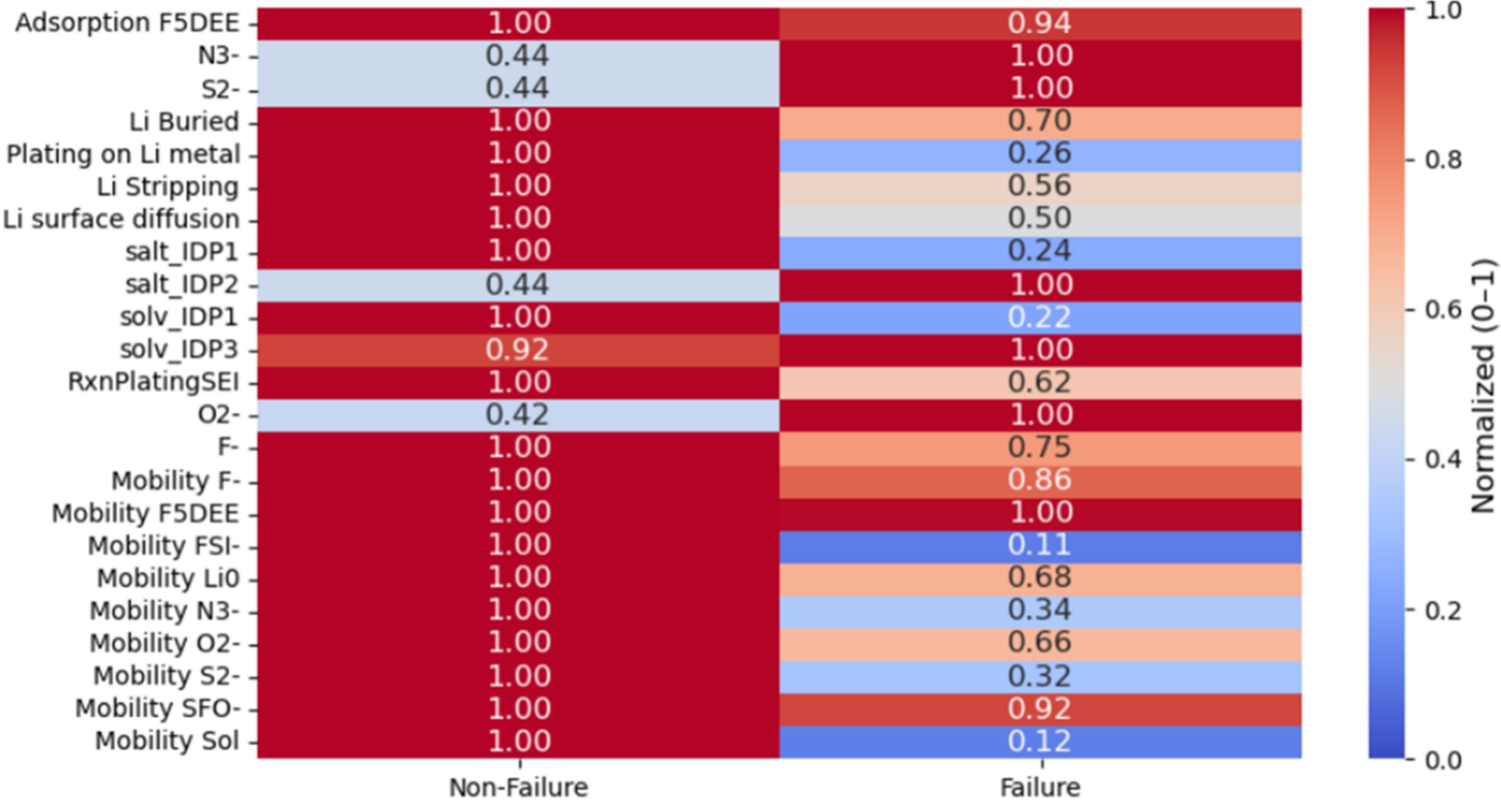
Heatmap of the mean normalized values for each parameter of the data set, comparing failure and nonfailure regions.

On the other hand, a strong increase of around 56–58% in the generation of N^3–^, S^2–^, O^2–^, and salt_IDP2 components is observed in the failure region, which mainly corresponds to the deep decomposition of LiFSI. Their accumulation is consistent with irreversible SEI thickening, side reactions, and loss of active lithium, all of which contribute to performance decay. Interestingly, solv_IDP3 (vinyl radical species), previously linked to high CE in the SHAP analysis, only decreases moderately in the failure zone, suggesting that its presence may delay degradation but is not sufficient to fully prevent it. Most other features also show lower average values in the failure region, reinforcing the idea that both chemical activity and ion mobility decline significantly once CE falls. Altogether, this binary comparison supports the trends observed in the SHAP and interval-based analyses and further emphasizes that early warning indicators of battery failure are marked by suppressed mobility of certain SEI components, reduced number of beneficial reaction events, and an increase in irreversible side product formation.

In terms of battery lifetime, almost 50% of the simulations in our data set reached failure in the range of 75–83 cycles, with a median at 78 cycles (Figure 7a). Based on our previous findings, Li-X pair-bonding interactions have the most strong influence on the CE. These pair interactions were used to train a variety of binary classifiers presented in the Computational Methods section (Logistic Regression, Random Forest, Gradient Boosting, XGBoost, and CatBoost) using a train–test split distribution of 60% and 40%, respectively. Employing the ROC-AUC criterion,^77^ the Random Forest Classifier achieved the highest value of 0.864, followed by XGBoost and CatBoost (the rest of the models are presented in the ROC curves in Figure S5). Nevertheless, considering all metrics, XGBoost emerged as the best model, due to its AUC values^78^ of 0.835 (second highest), while presenting the highest precision and recall scores (Table 5). The confusion matrix^79^ (Figure S7) shows that the model can successfully predict 76% of the failure and 84% of nonfailure battery performances, which indicates a reliable enough performance to be used in the SHAP analysis.

**Figure 7:**
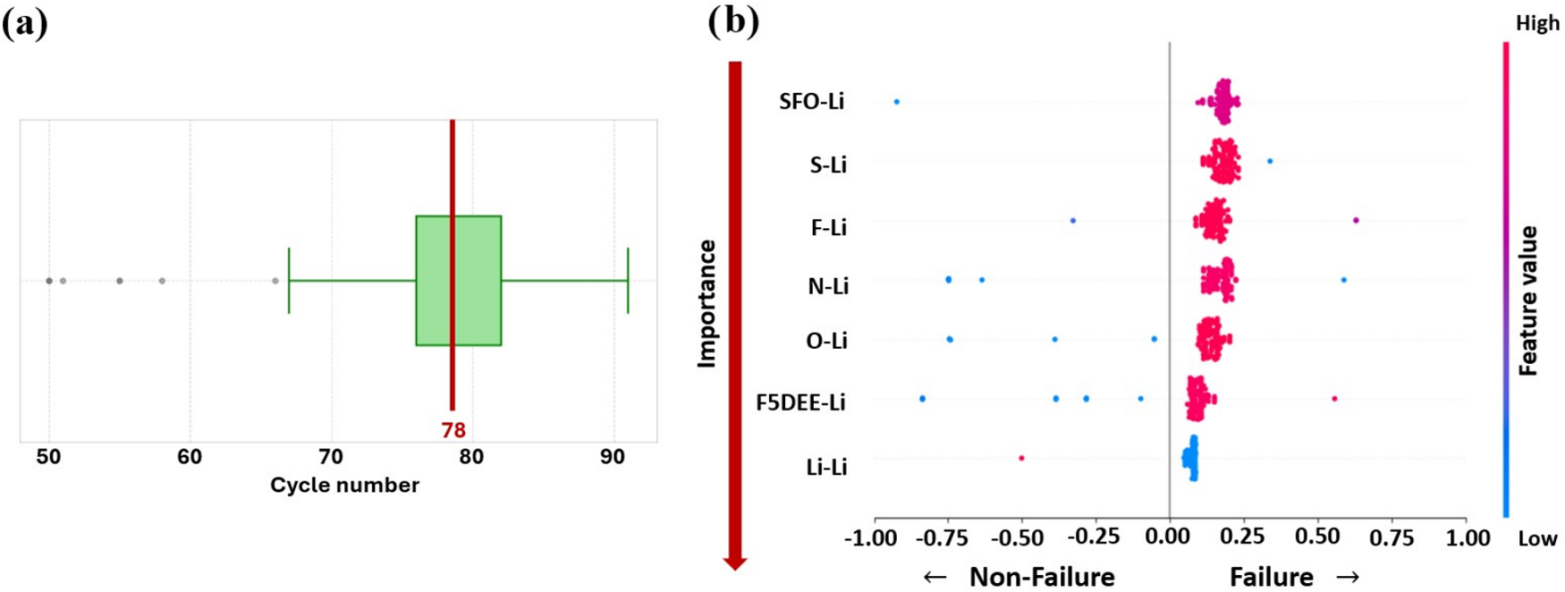
(a) Boxplot illustrates the predicted cycle number at which lithium battery failure is expected. (b) SHAP analysis of the XGBoost classifier for the binary classification of battery failure, where positive (red) SHAP values indicate features contributing to failure before 100 cycles.

**Table 5:** Accuracy, Precision, Recall, F1, and AUC Scores of the Best Classification Models Tested

model	accuracy	precision	recall	F1-score	AUC
Random Forest	0.7403	0.7272	0.7467	0.7368	0.8654
	Std: 0.0449	Std: 0.0566	Std: 0.0766	Std: 0.0484	Std: 0.0347
XGBoost	0.7988	0.7620	0.8533	0.8050	0.8346
	Std: 0.0429	Std: 0.0442	Std: 0.0722	Std: 0.0456	Std: 0.0332
CatBoost	0.7468	0.7000	0.8400	0.7636	0.8204
	Std: 0.0303	Std: 0.0403	Std: 0.0451	Std: 0.0266	Std: 0.0328
Gradient Boosting	0.7597	0.7375	0.7867	0.7613	0.7861
	Std: 0.0335	Std: 0.0367	Std: 0.0728	Std: 0.0378	Std: 0.0401
SVM	0.6623	0.6055	0.8800	0.7174	0.6559
	Std: 0.0287	Std: 0.0225	Std:0.0363	Std: 0.210	Std: 0.0551
Logistic Regression	0.7273	0.6542	0.9333	0.7692	0.6464
	Std: 0.0373	Std: 0.0360	Std: 0.0534	Std: 0.0283	Std: 0.0514

The SHAP results (Figure 7b) revealed that high pair-bonding interaction values of N–Li, F–Li, F5DEE-Li, and O–Li, along with low values of Li–Li, and moderate values of SFO-Li, correlated with premature failure. While SFO–Li interaction was the most important (consistent with previous results), the difference in mean absolute SHAP value across all Li-centered bonds was relatively small (0.44–0.19; Figure S6), suggesting that multiple Li-related interactions collectively contribute to failure rather than a single dominant bond. These results are related to the previous findings because high interactions of Li with the other chemical species generally correlate with lower CE. However, we did observe the exception that moderate SFO–Li interactions appear beneficial for stability. This suggests that despite SFO–Li’s negative impact on Li^+^ transport, it may help create a more cohesive and mechanically stable interphase. So, the balance of both effects must be studied in more detail.

## Conclusions

The integrated ab initio-kinetic Monte Carlo/machine learning workflow developed in this research demonstrates that data-driven surrogates can reproduce the CE landscape of Li metal cells and provide useful guidelines in electrolyte system design. The four-layer dense neural network delivered the highest predictive accuracy (RMSE ≈ 1.93%, *R* ^2^ ≈ 0.99), and SHAP analysis revealed that Li stripping, high decomposition of vinyl fluoride formation (solv_IDP3), and moderate LiF generation specially produced by adsorbent F5DEE fragmentation enhance the CE (moderate LiF contents stabilize the SEI and support Li^+^ transport, but excessive LiF elevates interfacial resistance, produce LiOH, and might encourage dendritic growth), whereas gathering of solv_IDP1, production of Li Buried, excessive production of dianionic salt fragment (salt_IDP2) together with accumulation of S^2–^, O^2–^, and N^3–^ species are correlated with CE reduction by thickening the SEI and reducing the transport of Li^+^. Furthermore, the results indicate that favoring further conversion of solv_IDP1 to solv_IDP3 without complete defluorination is advantageous. The mobility results indicate that dynamic reorganization of key inorganic SEI components (LiF, Li_2_O, Li_2_S, and Li_3_N) is essential, while the salt-derived fragments (SFO) should remain locally fixed to react in situ. Bayesian optimization (BO) analysis further showed that with the only exception of SFO–Li bonding, strong lithium pair interaction energies negatively impact battery performance, reducing CE, implying that electrolyte formulations should suppress excessive Li affinity toward fluorinated and oxygenated SEI chemical species, while selectively enhancing affinity for SFO-derived fragments.

Failure analysis through heatmaps reveals that once CE falls below 70%, mobility of key electrolyte and SEI species (FSI^–^ and solvent) drops, deep LiFSI reduction products surge, and uniform lithium plating ceases. Furthermore, it reveals that ∼50% of simulated cells cross the failure occurs between 75 and 83 cycles (median 78). The XGBoost classifier, validated by ROC-AUC = 0.835, predicts this transition reliably and attributes early failure to elevated N–Li, F–Li, F5DEE–Li, and O–Li interactions, moderate SFO–Li affinity, and low Li–Li interaction energies. These results align with previous results where the rise of N^3–^, S^2–^, O^2–^, and salt_IDP2 and with shortened mobility of FSI^–^ and solvent fragments, leading to reduction of Li^+^ transport across the interphase, loss of active lithium, and rapid CE decay.

In summary, based on the current electrolyte chemistry, the results prescribe general design principles that guidelines should enhance Coulombic efficiency and delay failure of lithium metal cells:

- Driving solvent decomposition toward vinyl fluoride-type species while limiting early intermediates.
- Maintaining LiF at moderate levels.
- Weaken most interfacial interactions except those linking Li to the salt decomposition products (SFO–Li), which should be tuned to an intermediate strength.
- Preserve mobility of FSI^–^, solvent remnants, and inorganic SEI phases during cycling,
- Monitor the interaction and mobility metrics as early predictors of efficiency loss.
- Sufficient mobility among key inorganic SEI components (e.g., LiF, Li_2_O, and Li_2_S) so they can reorganize within the interphase and evolve toward more stable and cohesive structures.
- Controlled solvent decomposition is favorable.
- Promote localized reactivity (low mobility, stronger interactions with Li ions) of salt-derived intermediate species.

Among factors causing the sudden drop of CE, the most important are associated with a sharp decrease in the mobility of anion and intermediates from solvent decomposition, which reflects the importance of the SEI dynamics. In addition, a reduced number of beneficial reaction events and SEI thickening contribute to impeding healthy plating and stripping on the surface.

Regarding connections of the conclusions of this work to experimental observables, here we outline preliminary ideas that may involve both theory and experiments. Detailed experimental analysis of the SEI composition and evolution during cycling is urgently needed. This may comprise NMR, electrochemical impedance spectra (EIS), operando microscopy, and other spectroscopic data capable of tracking SEI morphology and chemical features during cycling as well as interfacial transport. Aspects that need further understanding include ionic and electronic conductivities in basic SEI blocks as functions of their degree of crystallinity. The role of the SEI organic blocks and decomposition fragments in electron and ion transfer needs further exploration. Regarding the mobility of the SEI blocks during aggregation processes, developing techniques such as computed tomography for tracking SEI morphology should be at least an initial step.

Finally, we note that the basic AI-kMC/ML scheme presented here can be significantly enhanced. First, cathode degradation would modify the quality and composition of the ionic currents flowing to the anode side during cycling, known as cross-talk effects. Addressing the cathode side and its own interfacial and bulk degradation chemistry and physics starts at the AI/kMC level, followed by the combined AI-kMC/ML analysis. This requires first a modification of the AI-kMC model incorporating the cathode interfacial reactions and the model of the full cell. Then, a similar approach, as presented here, can be extended to include the cathode side, thus including additional factors that may influence the life of the complete cell. Another aspect derived from the work presented here is the extension of this work to other electrolyte chemistries. This can be done by following the workflow discussed in the AI-kMC work,^9^ which involves characterization of the ab initio thermodynamics and kinetics parameters of the new electrolyte chemistry prior to running the kMC algorithm, followed by the ML implementation shown in this study.

## Supplementary Material



## Notes

Heatmap of the mean absolute values of reaction features comparing equal size cycle intervals (Figure S1); heatmap of the mean normalized values of reaction features comparing equal size cycle intervals (Figure S2); heatmap of the mean absolute values of each feature, comparing equal size CE intervals (Figure S3); heatmap of the mean absolute values for each parameter of the data set, comparing failure and nonfailure regions (Figure S4); ROC Curves for different classifier models, predicting failure of simulated battery (Figure S5); average absolute SHAP values for the pairwise bonding interactions between Li and different SEI species (Figure S6); confusion matrix corresponding to the XGBoost classifier (Figure S7); and main thermodynamics and kinetics parameters used in this work (Tables S1 and S2) (PDF)
