# Supplementary material for: Evolution and Degradation Patterns of Electrochemical Cells Based on the Analysis of Interfacial Phenomena at Li Metal Anode/Electrolyte Interfaces
Source: J Phys Chem C Nanomater Interfaces. 2025 Aug 7;129(33):14687–700. doi: 10.1021/acs.jpcc.5c04292 (PMC12376106; doi:10.1021/acs.jpcc.5c04292)
Supplement: Supplementary file 1 [file jp5c04292_si_001.pdf]

Supporting Information for:

Evolution and Degradation Patterns of Electrochemical Cells based on Analysis of Interfacial Phenomena at Li Metal Anode/Electrolyte Interfaces

Carlos H. Guerrero Navarro[1] and Perla B. Balbuena[1,2,3]*
[1]Department of Chemical Engineering, [2]Department of Chemistry, [3]Department of Materials Science and Engineering, Texas A&M University, College Station, TX 77843

*e-mail: balbuena@tamu.edu

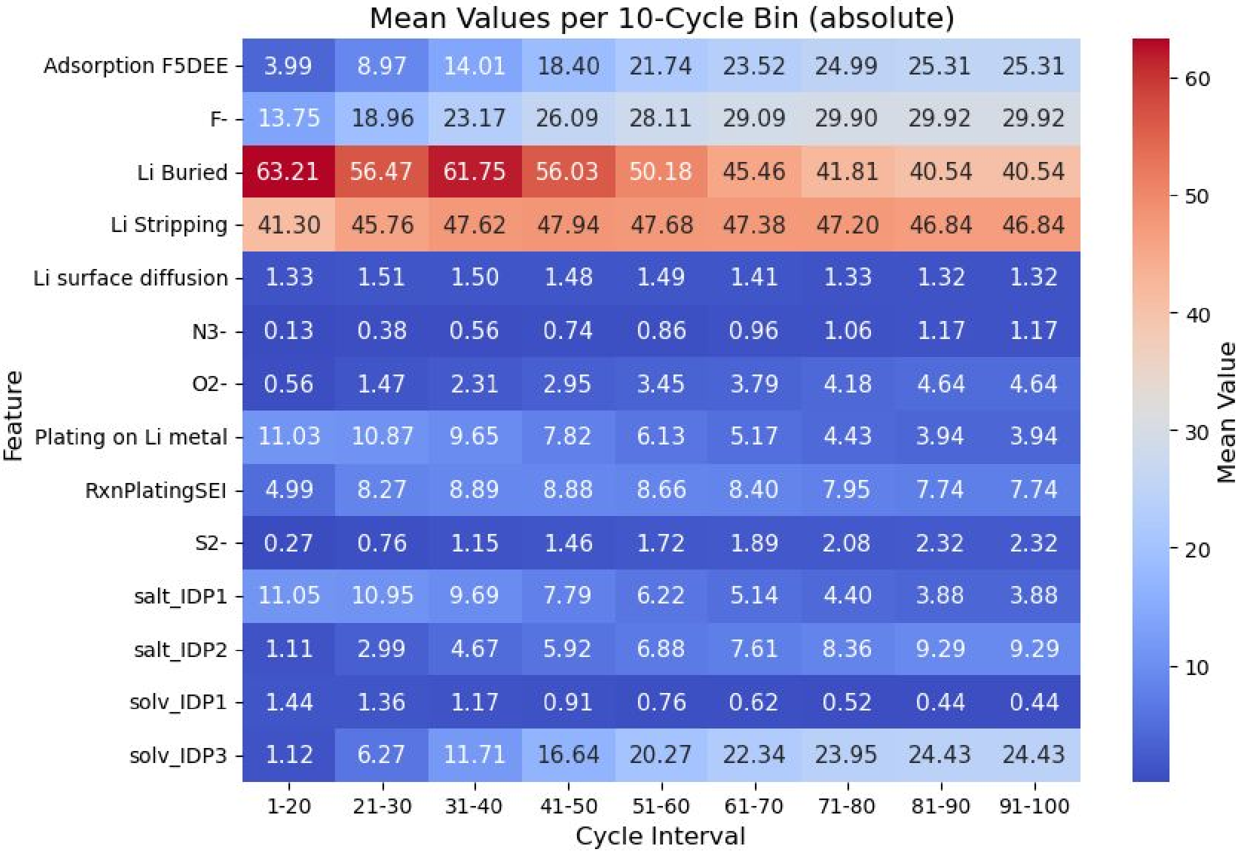

Figure S1. Heatmap of the mean absolute values of reaction features, comparing intervals of charge and discharge cycles.

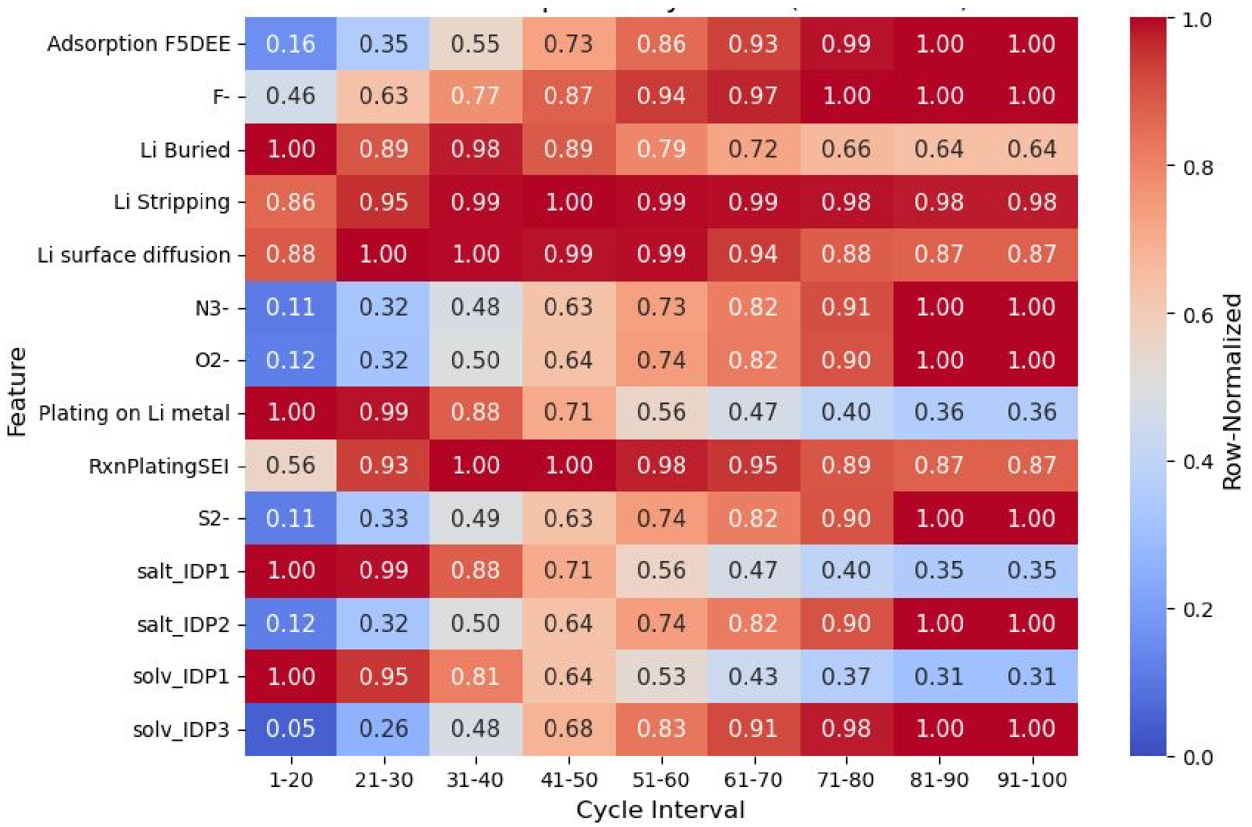

Figure S2. Heatmap of the mean normalized values of reaction features, comparing intervals of charge and discharge cycles.

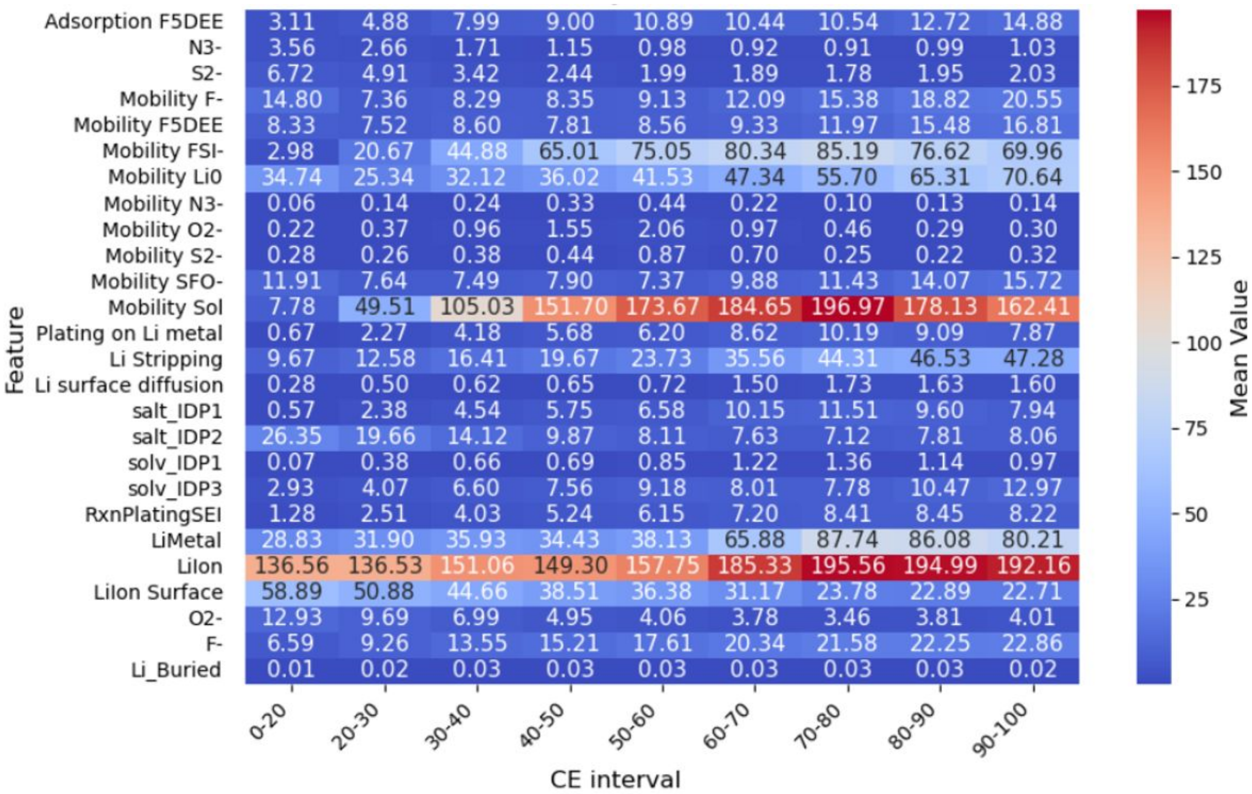

Feature	0-20	20-30	30-40	40-50	50-60	60-70	70-80	80-90	90-100
Adsorption F5DEE	3.11	4.88	7.99	9.00	10.89	10.44	10.54	12.72	14.88
N3-	3.56	2.66	1.71	1.15	0.98	0.92	0.91	0.99	1.03
S2-	6.72	4.91	3.42	2.44	1.99	1.89	1.78	1.95	2.03
Mobility F-	14.80	7.36	8.29	8.35	9.13	12.09	15.38	18.82	20.55
Mobility F5DEE	8.33	7.52	8.60	7.81	8.56	9.33	11.97	15.48	16.81
Mobility FSI-	2.98	20.67	44.88	65.01	75.05	80.34	85.19	76.62	69.96
Mobility Li0	34.74	25.34	32.12	36.02	41.53	47.34	55.70	65.31	70.64
Mobility N3-	0.06	0.14	0.24	0.33	0.44	0.22	0.10	0.13	0.14
Mobility O2-	0.22	0.37	0.96	1.55	2.06	0.97	0.46	0.29	0.30
Mobility S2-	0.28	0.26	0.38	0.44	0.87	0.70	0.25	0.22	0.32
Mobility SFO-	11.91	7.64	7.49	7.90	7.37	9.88	11.43	14.07	15.72
Mobility Sol	7.78	49.51	105.03	151.70	173.67	184.65	196.97	178.13	162.41
Plating on Li metal	0.67	2.27	4.18	5.68	6.20	8.62	10.19	9.09	7.87
Li Stripping	9.67	12.58	16.41	19.67	23.73	35.56	44.31	46.53	47.28
Li surface diffusion	0.28	0.50	0.62	0.65	0.72	1.50	1.73	1.63	1.60
salt_IDP1	0.57	2.38	4.54	5.75	6.58	10.15	11.51	9.60	7.94
salt_IDP2	26.35	19.66	14.12	9.87	8.11	7.63	7.12	7.81	8.06
solv_IDP1	0.07	0.38	0.66	0.69	0.85	1.22	1.36	1.14	0.97
solv_IDP3	2.93	4.07	6.60	7.56	9.18	8.01	7.78	10.47	12.97
RxnPlatingSEI	1.28	2.51	4.03	5.24	6.15	7.20	8.41	8.45	8.22
LiMetal	28.83	31.90	35.93	34.43	38.13	65.88	87.74	86.08	80.21
LiIon	136.56	136.53	151.06	149.30	157.75	185.33	195.56	194.99	192.16
LiIon Surface	58.89	50.88	44.66	38.51	36.38	31.17	23.78	22.89	22.71
O2-	12.93	9.69	6.99	4.95	4.06	3.78	3.46	3.81	4.01
F-	6.59	9.26	13.55	15.21	17.61	20.34	21.58	22.25	22.86
Li_Buried	0.01	0.02	0.03	0.03	0.03	0.03	0.03	0.03	0.02

Figure S3. Heatmap of the mean absolute values of each feature, comparing equal size CE intervals.

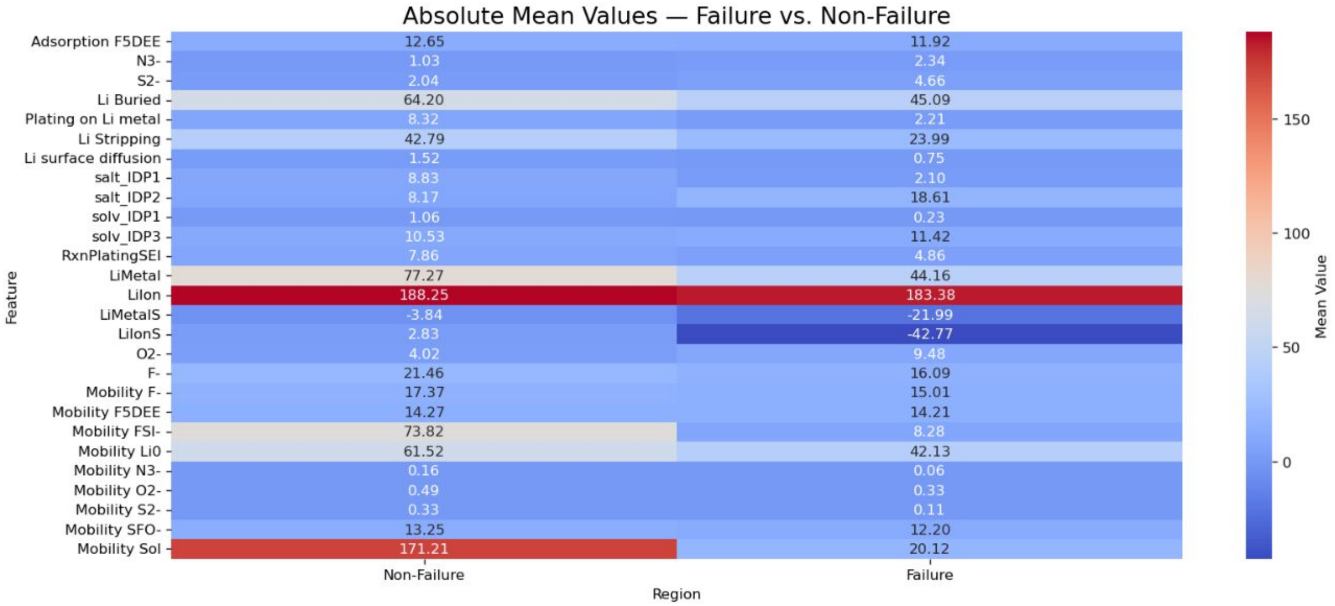

Figure S4. Heatmap of the mean absolute values for each parameter of the dataset, comparing failure and non-failure regions.

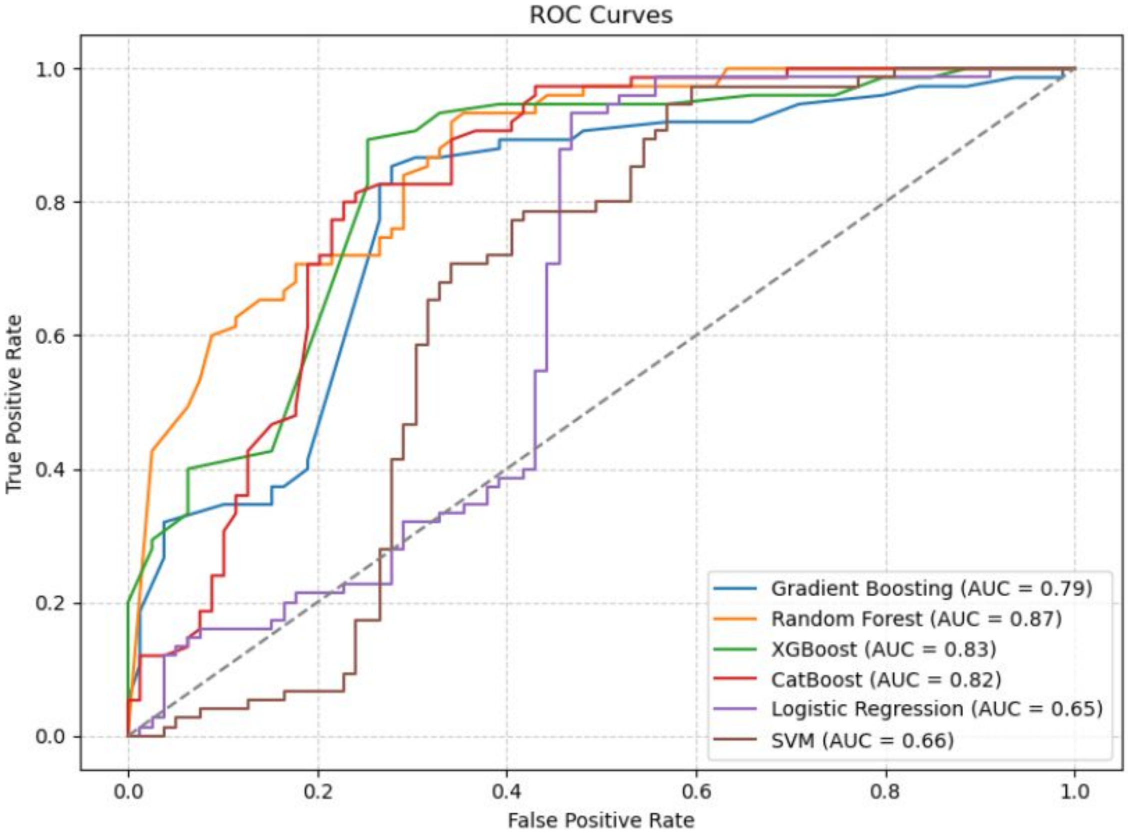

Figure S5. ROC Curves for different classifier models, predicting failure of battery simulation.

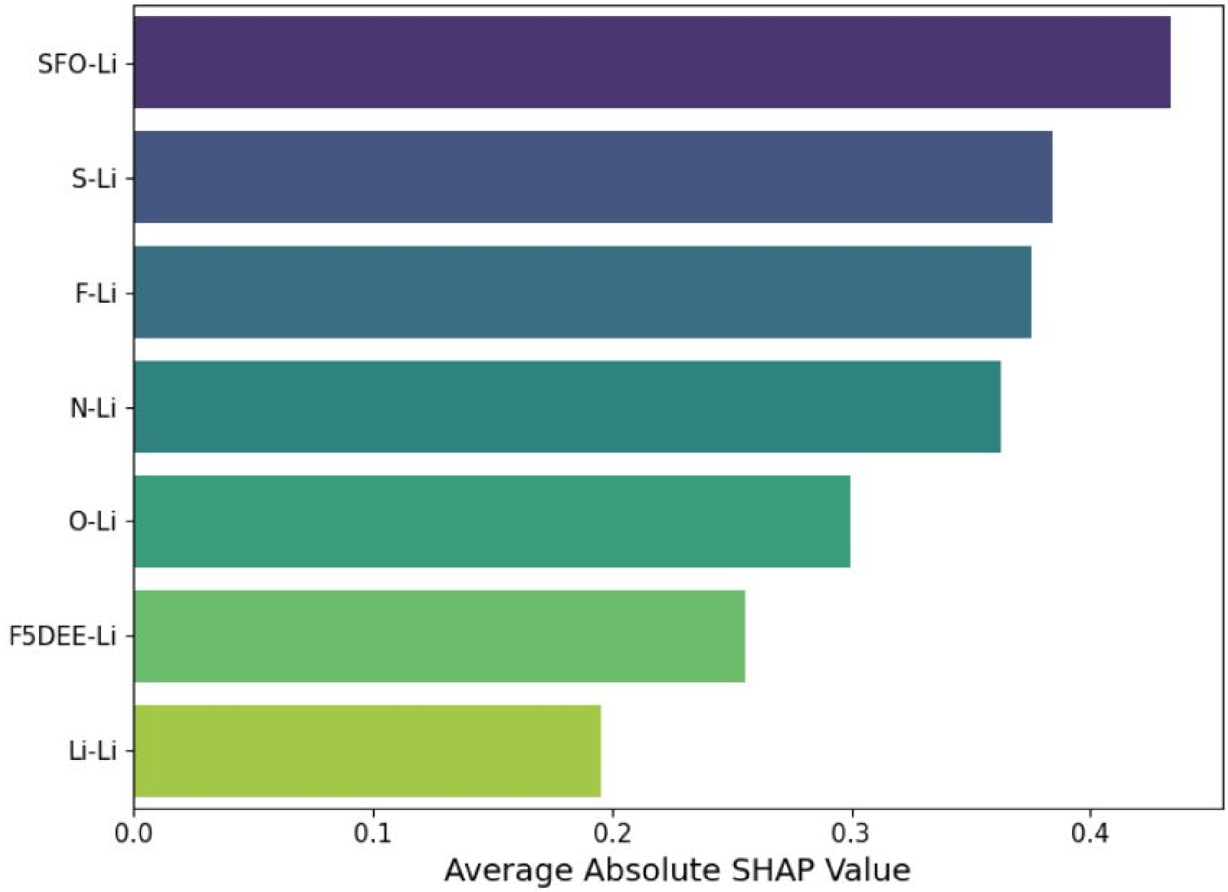

Figure S6. Average absolute SHAP values for the pairwise bonding interactions between lithium and different species in the SEI, obtained using an XGBoost classifier model

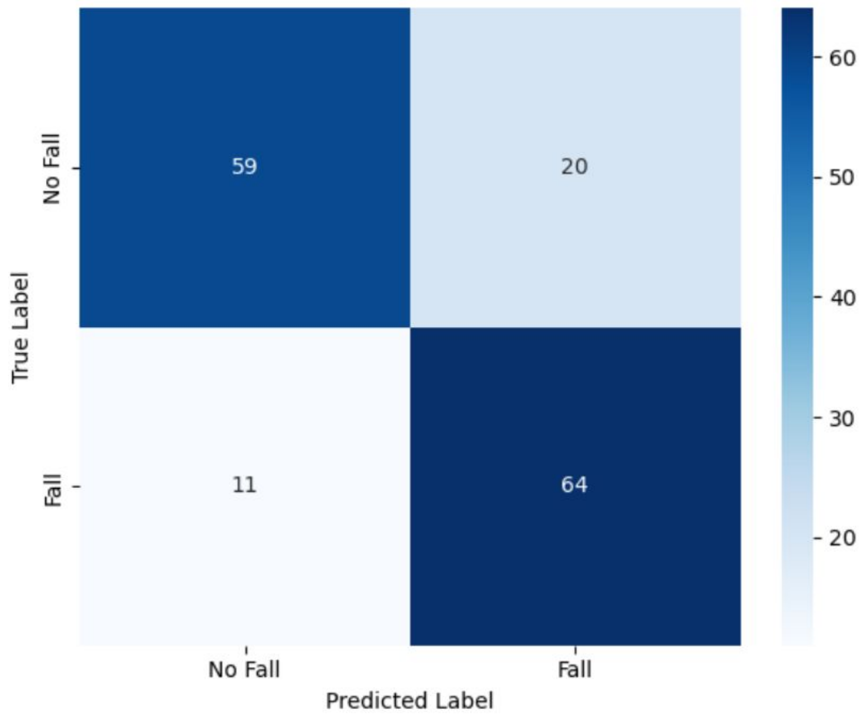

Figure S7. *Confusion matrix corresponding to the XGBoost classifier, evaluated on 40% of the simulation data.*

Table S1. AI-kMC parameters for decomposition reactions. k0: Arrhenius pre-exponential frequency factor. Ea: activation energy. E0: reference potential.

Reaction	$k0\ (s^{-1})$	$Ea\ (eV)$	$E0\ (V)$
Plating on Li metal	$1.0x10^{-3}$	0.0	-3.04
Plating over SEI	$1.0x10^{-3}$	0.0	-3.04
Salt Decom. Group 2	$1.0x10^{-5}$	0.0	-3.04
Solvent Decom. Group 1	$2.0x10^{-5}$	0.0	-3.04
Solvent Decom. Group 3	$1.0x10^{11}$	0.364	-3.04

Table S2. *A*I-kMC parameters for mobility reactions. k0: Arrhenius pre-exponential frequency factor. σ: fraction of sites available for diffusion. α: charge transfer coefficient indicating electrochemical sensitivity. E0: reference potential.

Reaction	$k0\ (s^{-1})$	σ	α	$E0\ (V)$
Li Stripping	$5.625x10^{1}$	1.0	0.0	0.0
Li Surface Diffusion	$5.625x10^{-1}$	1.0	0.0	-3.04
SFO Diffusion	$1.0x10^{11}$	1.0	0.0	0.0
F5DEE Diffusion	$1.0x10^{11}$	1.0	0.0	0.0
Li_3N Diffusion	$1.0x10^{11}$	1.0	0.0	0.0
LiF Diffusion	$1.0x10^{11}$	1.0	0.0	0.0
Li_2O Diffusion	$1.0x10^{11}$	1.0	0.0	0.0
Li_2S Diffusion	$1.0x10^{11}$	1.0	0.0	0.0
